# Miniaturized Portable Total Phosphorus Analysis Device Based on Photocatalytic Reaction for the Prevention of Eutrophication

**DOI:** 10.3390/mi12091062

**Published:** 2021-08-31

**Authors:** Dong Geon Jung, Maeum Han, Seung Deok Kim, Soon Yeol Kwon, Jin-Beom Kwon, Junyeop Lee, Seong Ho Kong, Daewoong Jung

**Affiliations:** 1 Advanced Mechatronics R&D Group, Korea Institute of Industrial Technology (KITECH), Daegu 42994, Korea; jdg8609@kitech.re.kr (D.G.J.); jinbum0301@kitech.re.kr (J.-B.K.); leejy@kitech.re.kr (J.L.); 2 School of Electronic and Electrical Engineering, Kyungpook National University, Daegu 41566, Korea; mehan@knu.ac.kr (M.H.); ksd5683@gmail.com (S.D.K.); k.soonyeol@knu.ac.kr (S.Y.K.)

**Keywords:** total phosphorus, photocatalytic reaction, eutrophication, MEMS, sensor

## Abstract

Phosphorus (P) is one of the most important elements in the aquatic ecosystem, but its overuse causes eutrophication, which is a serious issue worldwide. In this study, we developed a miniaturized portable total phosphorus (TP) analysis device by integrating a TP sensor with a photocatalyst to pretreat analyte and optical components (LED and photodetector) to measure the absorbance of the blue-colored analyte for real-time TP monitoring and prevention of eutrophication. The size of the miniaturized portable TP analysis device is about 10.5 cm × 9.5 cm × 8 cm. Analyte-containing phosphorus was pretreated and colored blue by colorizing agent as a function of the phosphorus concentration. Absorbance of the blue-colored analyte was estimated by the LED and the photodetector such that the phosphorus concentration was quantitatively measured. This device can obtain a wide linear response range from 0.5 mg/L to 2.0 mg/L (R^2^ = 0.97381), and its performance can be improved by increasing the intensity of the UV light emitted from the LED array. Consequently, the performance of this miniaturized portable TP analysis device was found to be similar to that of a conventional TP analysis system; thus, it can be used in automated in situ TP analysis.

## 1. Introduction

Phosphorus (P) is the most important component of living matter, such as DNA, cell membranes, enzymes, bones, and adenosine triphosphate (ATP). However, the eutrophication caused by its overuse has become a worldwide issue over the last decades. Various human activities, including agriculture, wastewater, urban expansion, and industry, are the main factors of phosphorus overuse. Etymologically, eutrophication means “well nourished.” It occurs as a result of the oversupply of nutrients such as phosphorus and nitrogen in an aquatic ecosystem and leads to algal bloom [1,2,3,4,5,6,7,8,9,10,11,12,13,14]. A eutrophicated aquatic ecosystem or one suffering from algal bloom can disrupt the planktonic stand structure. Toxins can also be produced by the proliferation of unwanted algae, such as green algae and cyanobacteria, and can have a negative impact on human health (e.g., stomachaches, rashes, and more serious health issues). Algal blooms induce quite a high amount of oxygen consumption, thus depriving fish and shellfish.

Accordingly, many studies worldwide have reported the monitoring and regulation of total phosphorus (TP) concentration in aquatic ecosystems to prevent the abovementioned serious problems caused by eutrophication. In particular, there exists a growing need for the development of a real-time TP analysis system. Some requirements, such as portability, affordability, and short analysis time, must be satisfied to develop this kind of system.

**Figure 1:**
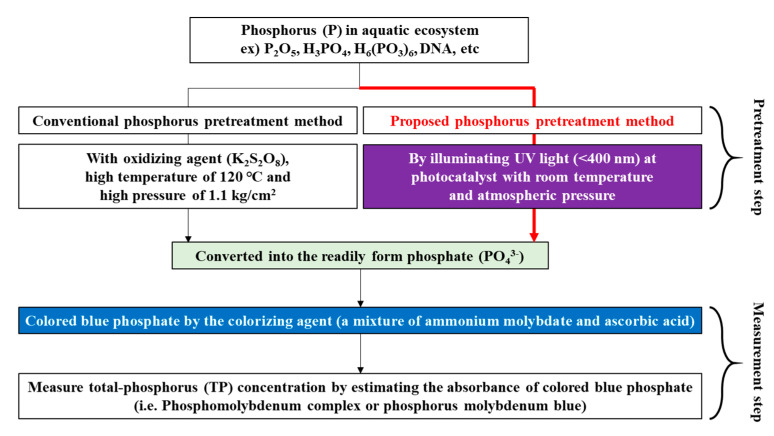
Principle of TP analysis utilizing the conventional and proposed methods.

The conventional TP analysis system is mainly used in laboratories and cannot monitor TP concentration in real time because of disadvantages including bulky size, expensiveness, and long analysis time. The TP analysis procedure consists of the pretreatment and measurement steps shown in Figure 1. The pretreatment step, which is the most important step in the TP analysis procedure, is performed under high temperature (>120 °C) and pressure (>1.1 kg cm^−2^) using a conventional pretreatment device (i.e., autoclave) [15,16,17,18,19,20,21]. The phosphorus in an aquatic ecosystem is combined with various inorganic/organic materials and exists in diverse forms; thus, it must be converted into the readily analyzable form phosphate (PO_4_^3−^) by the pretreatment step to achieve an accurate TP analysis. After the pretreatment step, the pretreated phosphorus is converted into phosphate (PO_4_^3−^) and colored blue by the prepared colorizing agent. The prepared colorizing agent selectively colors the decomposed phosphate in the pretreated analyte blue (phosphorus is converted into phosphate); thus, the colorizing agent plays the role of TP concentration indicator. Finally, the TP concentrations in the analyte are measured by estimating the absorbance of the blue-colored analyte.

Besides the colorimetry method, there are various methods for measuring TP concentration quantitatively, such as the gravimetric method, volumetric method, and potentiometric titration [22]. These methods have been extremely popular for decades due to their economical utility, convenience, and large measurement range. Recently, a TP measuring method utilizing spectrophotometry, which could overcome the disadvantages of traditional methods of measuring TP (complex manual for operation, poor stability, and low sensitivity), has been receiving attention. Development of TP measuring methods utilizing optical properties of materials and the detection principles of spectrophotometry—including the use of chemosensor, test strip, photoelectric colorimetry, automated and intelligent flow injection analysis, microfluidics, and some characteristic spectral techniques—has accelerated. Utilization of test strips for measuring TP is one of the representative examples. A mixture of ammonium molybdate and ascorbic acid embedded in a test strip reacts with the pretreated phosphorus (phosphate, PO_4_^3−^), and phosphorus molybdenum blue is formed. TP concentration is quantitatively measured by comparing the depth of the blue color on the test strip. This TP measuring method with test strips is widely used in the analysis of TP because of its fast response time and convenience; thus, it is suitable for on-site measurement. However, the TP measuring method with test strips has some limitations, as follows: (1) poor precision due to observation with the naked eye; and (2) TP measurement is not entirely on site, because the pretreated phosphorus (phosphate, PO_4_^3−^) should be prepared in advance. As mentioned above, the need for development of real-time TP analysis is growing; thus, a novel TP pretreatment and TP measuring method must be developed to meet this need by developing a portable device with good sensitivity and precision, high reliability, and so on.

Many studies on portable TP analysis systems have recently been conducted with the aim of preventing eutrophication or algal bloom. Developing an advanced pretreatment method without harsh conditions (high temperature and pressure) and measurement methods is fundamental to developing a portable TP analysis system based on cutting-edge manufacturing technologies, such as micro-electro-mechanical-system (MEMS), lab-on-a-chip (LOC), and microfluidics [23,24,25,26,27,28,29,30,31,32,33,34,35,36,37,38,39]. However, most of these studies did not integrate all TP analysis procedures into a single portable device. Furthermore, various disadvantages, including a complex structure, difficulties in fabrication, and high cost, have been observed.

One promising advanced pretreatment method without harsh conditions is to utilize a photocatalyst and the photocatalytic reaction [38]. The photocatalytic reaction accelerates the chemical reaction by utilizing a catalyst and light with a wavelength corresponding to the energy band gap (*E_g_*) of the catalyst. An artificial light source with an ultraviolet (UV) wavelength component is frequently used to generate the photocatalytic reaction because commercial photocatalysts (e.g., TiO_2_, ZnO, and WO_3_) have a range of absorbance wavelengths from 350 to 400 nm. TiO_2_ is frequently utilized as the photocatalyst material because of its high activity, low cost, non-toxicity, and chemical inertness. When a UV light of 365 nm wavelength illuminates the photocatalyst (TiO_2_), a hydroxyl radical (·OH) with an excellent oxidation strength is generated, and this hydroxyl radical decomposes various compounds in the water [40,41,42,43,44,45,46,47,48,49,50,51,52,53,54].

By utilizing photocatalytic reaction, the phosphorus combined with various inorganic/organic materials can be easily decomposed (converted) into the readily analyzable form of phosphate (PO_4_^3−^) without high temperature and internal pressure. This pretreatment method enables the water-quality-monitoring procedure to be integrated into and miniaturized on a portable system, because the photocatalytic-reaction-based pretreatment conditions under room temperature and atmospheric pressure provide freedom in design and fabrication.

Next, pretreated phosphorus, which is converted into phosphate (PO_4_^3−^) as a readily analyzable form, is colored blue, and the depth of blue color of the analyte must be measured quantitatively by utilizing an advanced TP measuring method, in order to overcome poor precision due to observation with the naked eye. Blue-colored analyte absorbs light with the desired wavelength, and its absorbance is generally measured with a UV–Vis spectrometer. The UV–Vis spectrometer is frequently used in analysis fields utilizing spectrophotometry, but it is not suitable for on-site measurement because of its bulky size, complex manual for operation, and expensiveness. To solve these problems, a photoelectric strategy which converts optical properties of the analyte to electrical characteristics is proposed in this study, utilizing optical components such as an LED and a photodetector. This TP measuring method utilizing a photoelectric strategy not only improves the detection efficiency but also makes the detection process intelligent, and the detection results can be visualized.

In this study, a miniaturized portable TP analysis device utilizing a TP sensor with a photocatalyst, an LED, and a photodetector was fabricated and characterized for monitoring TP concentration and preventing eutrophication. All parts—which include pretreatment, mixing, and measurement parts for analyzing TP—were integrated into a miniaturized portable TP analysis device by utilizing MEMS, LOC, and three-dimensional (3D) printing technologies. In particular, the photocatalytic-reaction-based pretreatment method is very important to develop a miniaturized portable TP analysis equipment. TiO_2_ is utilized as the photocatalyst in this study, considering its high activity, low cost, non-toxicity, and chemical inertness. An LED array with peak wavelengths of 365 nm and 880 nm and a photodetector were installed in a miniaturized portable TP analysis device with a fabricated small-sized TP sensor to activate the photocatalytic reaction and measure the absorbance of the blue-colored analyte containing pretreated phosphate. The UV light intensity emitted from the LED array was increased to enhance the photocatalytic reaction efficiency in comparison with the conventional pretreatment method utilizing an autoclave. After photocatalytic-reaction-based pretreatment, the absorbance of the blue-colored analyte as a function of the TP concentration was estimated by measuring photodetector output signals. This means that the entire process for TP analysis can be easily conducted without time and location restrictions by utilizing a miniaturized portable TP analysis device, as described in this paper.

## 2. Proposed TP Analysis Procedure Applied to a Miniaturized Portable TP Analysis Device

As mentioned in the Introduction, monitoring the TP concentration of an aquatic ecosystem in real time is very important because phosphorus overuse can induce eutrophication or algal bloom.

**Figure 2:**
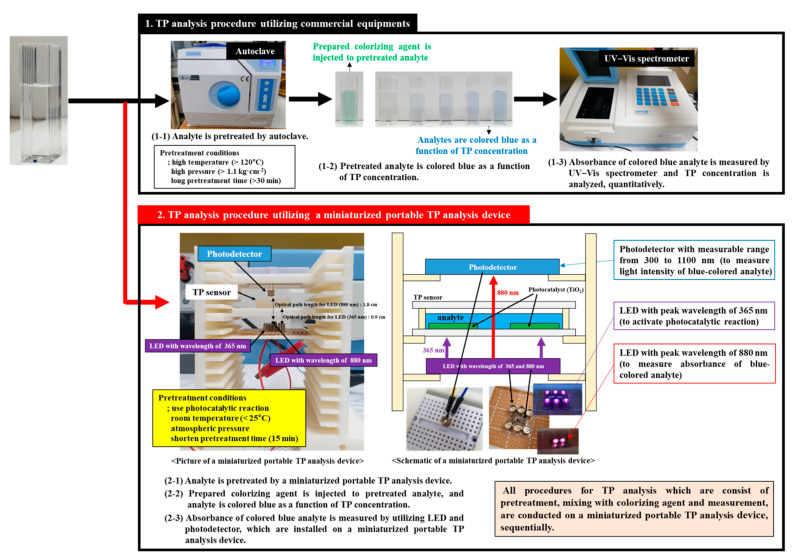
TP analysis procedures utilizing commercial equipment (i.e., autoclave and UV–Vis spectrometer) and the miniaturized portable TP analysis device described in this work.

Figure 2 shows TP analysis procedures utilizing commercial equipment (i.e., autoclave and UV–Vis spectrometer) and a miniaturized portable TP analysis system, respectively. The TP analysis procedure complied with the “Standard Methods (APHA Method 4500-P: Standard Method for Examination of Water and Wastewater).” Among the methods suggested in the “Standard Methods (APHA Method 4500-P),” the persulfate digestion and ascorbic acid methods were utilized herein to pretreat the analytes and measure the TP concentration, respectively [55]. In the conventional TP analysis procedure (step 1-1, 1-2, 1-3 in Figure 2), the pretreatment step was performed at a high temperature (>120 °C) and a high internal pressure (>1.1 kg cm^−2^) by utilizing an autoclave (step 1-1, Figure 2), which was bulky. Phosphorus in the water sample was converted into the readily analyzable form phosphate (PO_4_^3−^) through the pretreatment step, and phosphate (PO_4_^3−^) was selectively colored blue by a prepared colorizing agent (step 1-2, Figure 2). TP concentration was measured by estimating the absorbance of blue-colored phosphate (PO_4_^3−^) in the water sample (step 1-3, Figure 2). A UV–Vis spectrometer is used to measure the absorbance of blue-colored phosphate (PO_4_^3−^). Conventional TP analysis procedures utilizing commercial equipment (i.e., autoclave and UV–Vis spectrometer) can measure TP concentration accurately. However, it is not suitable for on-site and real-time TP analysis because the equipment used, such as the autoclave and UV–Vis spectrometer, has some disadvantage, including bulky size, expensiveness, complex manual for operation, and long analysis time. In addition, realizing the pretreatment method used in commercial equipment on a miniaturized TP analysis device with a portable size, which has physical and mechanical weaknesses, is difficult.

The TP analysis procedure utilizing the miniaturized portable device is comprised of two steps: pretreatment (steps 2-1 and 2-2 in Figure 2) and measurement (step 2-3 in Figure 2). All TP analysis procedures were conducted on the miniaturized portable TP analysis device sequentially. The pretreatment step was performed to convert phosphorus combined with various materials in the water sample into readily analyzable forms, such as phosphate (PO_4_^3−^). Unlike the conventional TP analysis procedure, the photocatalytic-reaction-based pretreatment method was applied herein for a miniaturized portable TP analysis device manufactured using the MEMS, LOC, and 3D printing technologies. The photocatalytic-reaction-based pretreatment method was performed under room temperature and atmospheric pressure; hence, it can be easily applied to a miniaturized portable TP analysis device. In addition, the UV light intensity emitted from the LED array was increased to improve the photocatalytic reaction efficiency. The measurement step is to estimate the concentration of pretreated and blue-colored phosphates (PO_4_^3−^) by characterizing their absorbance. As mentioned above, a photoelectric strategy which converts optical properties of the blue-colored phosphates (PO_4_^3−^) into electrical characteristics is utilized by installing optical components, such as an LED and a photodetector, in the miniaturized portable device [56]. 

The photocatalytic reaction is generated in the presence of a catalyst that accelerates the photoreaction, and various semiconductors have been used as photocatalysts (i.e., TiO_2_, ZnO, WO_3_, etc.). When a semiconductor absorbs the light with a wavelength corresponding to its energy band gap (*E_g_*), electron–hole pairs (EHPs) are generated. The generated EHPs migrate to the semiconductor surface and can reduce and oxidize the reactants which are absorbed by the semiconductor. This process is called the photocatalytic reaction. Among the various photocatalysts (e.g., TiO_2_, ZnO, WO_3_, etc.), titanium oxide (TiO_2_) was used herein as the catalyst for accelerating the photocatalytic reaction. TiO_2_ was discovered in 1791 from ilmenite, and its photoactivity was first noticed when it was used as white pigment in buildings in 1929. TiO_2_ exhibit high activity, low cost, non-toxicity, and chemical inertness. The photocatalytic activity of TiO_2_ is applied to an enormous range of fields, such as photocatalytic water-splitting, photocatalytic self-cleaning, purification of wastewater, photo-induced super hydrophilicity, photovoltaics, and so on. The most promising application of TiO_2_ as a photocatalyst is the photodegradation of various environmental pollutants, such as complex organic/inorganic compounds that turn into CO_2_, H_2_O, and harmless inorganic anions, respectively. Studies on TiO_2_ as a catalyst are ongoing worldwide. Despite the usefulness of TiO_2_ as a photocatalyst in the photodegradation of various environmental pollutants, there are two major disadvantages that limit its photocatalytic reaction efficiency: (1) only 3–5% of the whole solar spectrum (UV region) is used efficiently for generating the photocatalytic reaction due to the large band-gap (*E_g_*) of TiO_2_, and (2) short lifetime of photo-generated EHPs due to the recombination. To overcome these disadvantages, several studies have been conducted to enhance the photocatalytic activity of TiO_2_, including dye sensitization, doping with metals/non-metals, and coupling with carbonaceous nanoscale materials (carbon nanotubes and graphene) [57,58,59]. However, utilizing TiO_2_ as a photocatalyst and an artificial UV light on a portable TP analysis device is the most promising method, because the photocatalytic-reaction-based pretreatment procedure is conducted under room temperature and atmospheric pressure, resulting in freedom of design and fabrication. 

**Figure 3:**
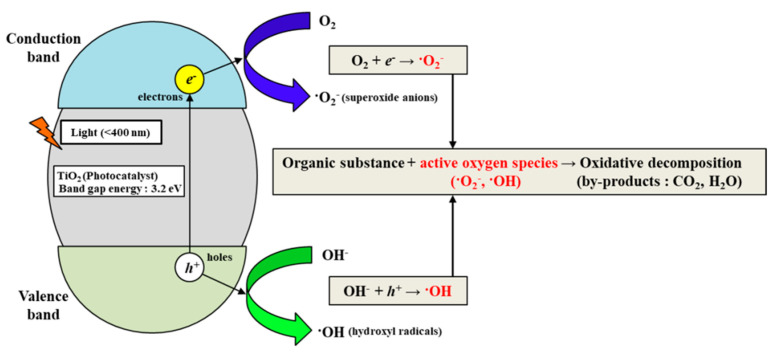
Mechanism of water pollutants’ decomposition utilizing photocatalyst (TiO_2_).

In this paper, the photocatalytic activity of TiO_2_ is utilized to analyze TP concentration. Figure 3 shows the mechanism of the decomposition of water pollutants (various compounds containing phosphorus) utilizing a photocatalyst (TiO_2_). By illuminating light with a wavelength corresponding to the energy band gap (i.e., approximately 3.2 eV) of TiO_2_, EHPs are generated, and the photocatalytic reaction occurs on the TiO_2_ surface. The generated EHPs on the TiO_2_ surface and the hydroxyl ions in the water generate hydroxyl radicals (∙OH) with a strong oxidation strength. The photocatalytic-reaction-induced hydroxyl radicals (∙OH) with a strong oxidation strength rapidly decompose water pollutants (various compounds containing phosphorus). As a result, phosphorus combined with various materials is converted into phosphate (PO_4_^3−^). Phosphate (PO_4_^3−^) is colored blue (forms a phosphomolybdenum complex) by the prepared colorizing agent, and its concentration is measured quantitatively. 

## 3. Experiment

### 3.1. Preparation of Reagents for Characterizing the Miniaturized Portable TP Analysis Device

For characterizing a miniaturized portable TP analysis device, sodium glycerophosphate (C_3_H_7_Na_2_O_6_P) was employed as the typical phosphorus solution (analyte) and was prepared ranging from 0.5 to 2.0 mg/mL (by weight of P). Then, ascorbic acid (C_6_H_8_O_6_), ammonium molybdate ((NH_4_)_6_Mo_7_O_24_·4H_2_O), potassium antimony tartrate (KSbC_4_H_4_O_7_·1/2H_2_O), and H_2_SO_4_ were utilized to prepare the colorizing agent. Potassium persulfate (K_2_S_2_O_8_) was utilized as the oxidizing agent.

### 3.2. Design and Fabrication of a Miniaturized Portable TP Analysis Device

**Figure 4:**
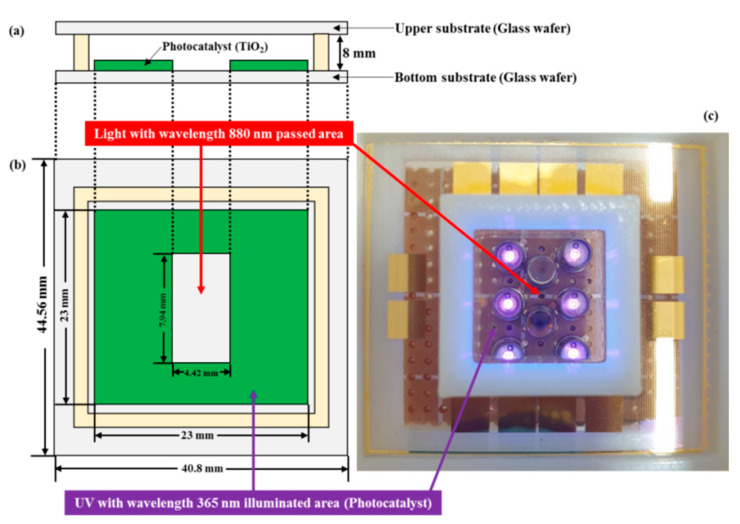
Schematic of the proposed TP sensor, (**a**) cross view and (**b**) top view, and (**c**) picture of the fabricated TP sensor.

**Figure 5:**
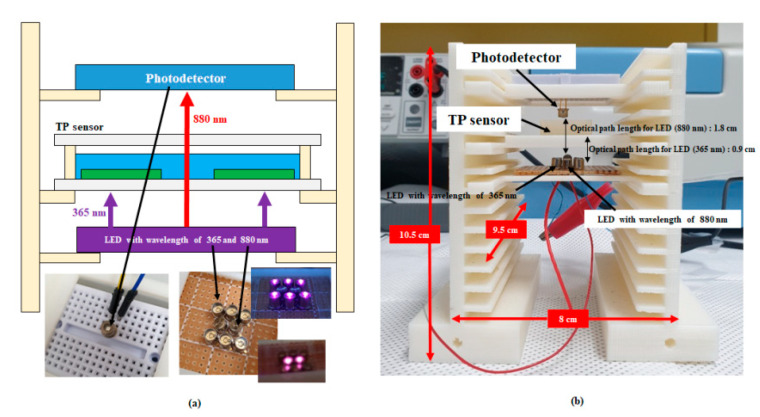
(**a**) Schematic and (**b**) picture of the miniaturized portable TP analysis device with the TP sensor.

Figure 4 and Figure 5 show schematics and pictures of the proposed TP sensor and a miniaturized portable TP analysis device with the fabricated TP sensor. The miniaturized portable TP analysis device consisted of a small-sized TP sensor, LEDs with 365 nm wavelength for activating the photocatalytic reaction and 880 nm wavelength for measuring the absorbance of the blue-colored analyte, and a photodetector with a measurable range from 300 to 1100 nm. The miniaturized portable TP analysis device measured the absorbance of the colorized analyte as a function of the TP concentration; hence, a transparent glass wafer was utilized for both the upper and bottom substrates. Portable MEMS-based sensors generally utilize silicon (Si) wafers because they are produced through well-developed manufacturing technologies (e.g., bulk and surface micro-machining). However, a Si wafer is opaque in the wavelength range of the UV to NIR region; hence, a glass wafer was utilized herein for both upper and bottom substrates.

**Figure 6:**
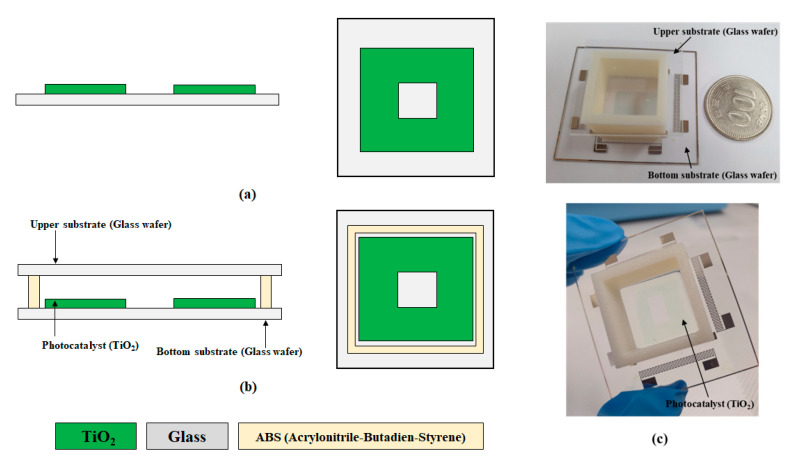
Fabrication process of the proposed TP sensor: (**a**) TiO_2_ (200 nm) deposition, (**b**) bonding upper/bottom substrates and reactive chamber (height: 8 mm), and (**c**) pictures of the fabricated TP sensor.

Figure 6a,b presents the fabrication procedure of the TP sensor which will be integrated into the miniaturized portable TP analysis device. MEMS technologies, such as photolithography and thin film deposition, were utilized to fabricate the proposed TP sensor. A reactive chamber was fabricated by utilizing a 3D printing technology to easily obtain the desired dimensions and structure.

Glass wafer was used for the upper and bottom substrates of the proposed TP sensor because of its transparency in the wavelength range from the UV to NIR region. A 200 nm-thick TiO_2_ (photocatalyst) was deposited by sputtering system (manufacturer: SORONA, model: SRN-110) and patterned by the lift-off process. To transmit the light emitted from the LED with a peak wavelength of 880 nm, TiO_2_ was not deposited at the center of the bottom substrate (Figure 3). Subsequently, a reactive chamber was fabricated by utilizing 3D printing equipment for easy fabrication and affordability. Finally, the fabricated reactive chamber and the upper and bottom substrates were bonded by an adhesive composed of a mixture of polydimethylsiloxane (PDMS) and curing agent (PDMS: curing agent = 10:1; curing temperature: 60 °C; and curing time: 4 h).

## 4. Results and Discussions

**Figure 7:**
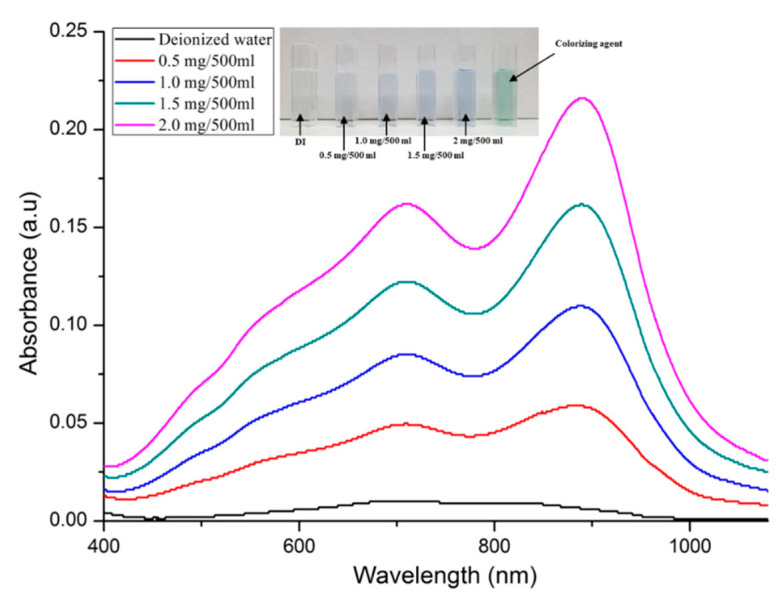
Measured absorbance as a function of the phosphate concentration after pretreatment step by autoclave.

Before characterizing the miniaturized portable TP analysis device, absorbance as a function of phosphate (PO_4_^3−^) concentration was measured using the un-pretreated phosphorus analyte (sodium glycerophosphate (C_3_H_7_Na_2_O_6_P)), the oxidizing agent (K_2_S_2_O_8_), and the colorizing agent (i.e., a mixture of ascorbic acid (C_6_H_8_O_6_), ammonium molybdate ((NH_4_)_6_Mo_7_O_24_·4H_2_O), potassium antimony tartrate (KSbC_4_H_4_O_7_·1/2H_2_O), and H_2_SO_4_). After the pretreatment process conducted using conventional equipment (i.e., autoclave), the analytes were colored blue by a prepared colorizing agent, as phosphorus was converted into phosphate (PO_4_^3−^) and formed a phosphomolybdenum complex. A UV–Vis spectrometer (BKV-1800PC, Bio Konvision Co., Ltd., Gyeonggi-do, Republic of Korea) was used to measure the absorbance of the blue-colored analyte (i.e., phosphomolybdenum complex). Figure 7 shows the analytes colored blue by a prepared colorizing agent and the measured absorbance as a function of the phosphate (PO_4_^3−^) concentration. The absorbances measured by the UV–Vis spectrometer were increased by increasing the phosphate (PO_4_^3−^) concentration. The maximum absorption wavelength was at 880 nm. As mentioned in the Introduction, the photoelectric strategy is applied to the miniaturized portable TP analysis device to easily and accurately obtain an electrical signal corresponding to the optical properties of blue-colored analytes. For these reasons, LED arrays with peak wavelengths of 365 and 880 nm and a photodetector (MTAPD-06, Marktech Optoelectronics, Latham, NY, USA) with a measurable range from 300 to 1100 nm were applied to the miniaturized portable TP analysis device. 

**Figure 8:**
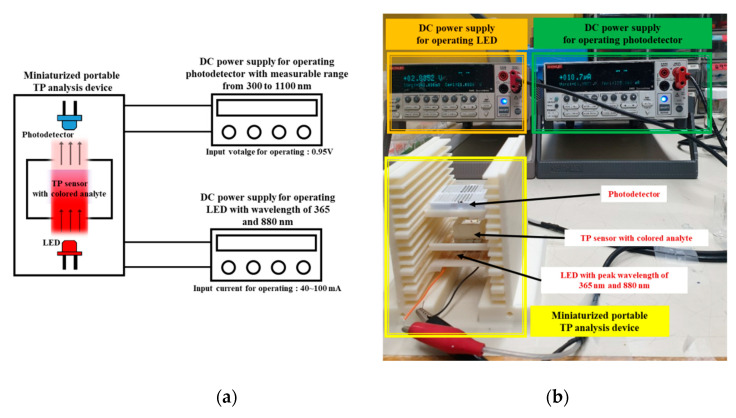
(**a**) Schematic diagram of the experimental setup for assessing the miniaturized TP analysis device and (**b**) picture of the experimental setup.

Next, the miniaturized portable TP analysis device with a TP sensor was assessed by utilizing the experimental setup in Figure 8. The un-pretreated phosphorus analyte (sodium glycerophosphate (C_3_H_7_Na_2_O_6_P)) at 2 mg/500 mL and the oxidizing agent (K_2_S_2_O_8_) were injected into the fabricated TP sensor. Subsequently, a UV LED (OL375KFF, ODTech Co., Ltd., Cheonbuk, Korea) array was turned on to generate the photocatalytic-reaction-based pretreatment process by applying an operating current of 40 mA for 5–30 min. Note that an operating current of 20–40 mA was suggested as a typical value in the manufacturer’s manual. The pretreatment process time utilizing the miniaturized portable TP analysis device was limited to 30 min because the conventional pretreatment process utilizing the autoclave is normally conducted for 30 min under the conditions of high temperature (>120 °C) and internal pressure (>1.1 kg cm^−2^). The miniaturized portable TP analysis device herein was developed to shorten the analysis time and avoid harsh analysis conditions.

**Figure 9:**
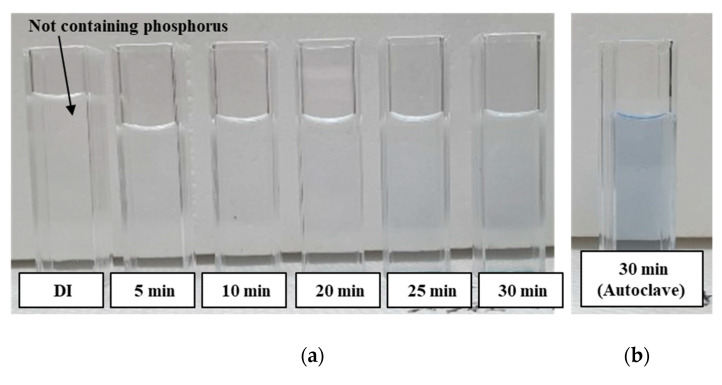
(**a**) Pretreated and colorized analytes (2 mg/500 mL) using a miniaturized portable TP analysis device with pretreatment times (UV operating current: 40 mA), and (**b**) pretreated and colorized analytes (2 mg/500 mL) using the autoclave.

After pretreatment through the photocatalytic reaction, the pretreated analytes were then colored blue as a function of the pretreatment time. This means that the phosphorus is converted into the readily analyzable form phosphate (PO_4_^3−^), and phosphomolybdenum complex is formed by the reaction with the prepared colorizing agent. The pretreated analytes were colored a deeper blue when the pretreatment time was increased, as shown in Figure 9. That is, the larger the amount of phosphorus decomposed as phosphate (i.e., phosphorus is converted into phosphate), the higher the amount of phosphomolybdenum complex formed because of the longer photocatalytic-reaction-based pretreatment time. Deionized (DI) water was not colored blue because it did not contain phosphorus. However, the analytes pretreated for 5 to 30 min by the miniaturized portable TP analysis device were not fully colored blue compared to the analytes pretreated by the autoclave, indicating that these analytes were not perfectly pretreated. The UV light intensity was increased to enhance the photocatalytic reaction efficiency and improve the pretreatment performance of the miniaturized portable TP analysis device.

**Figure 10:**
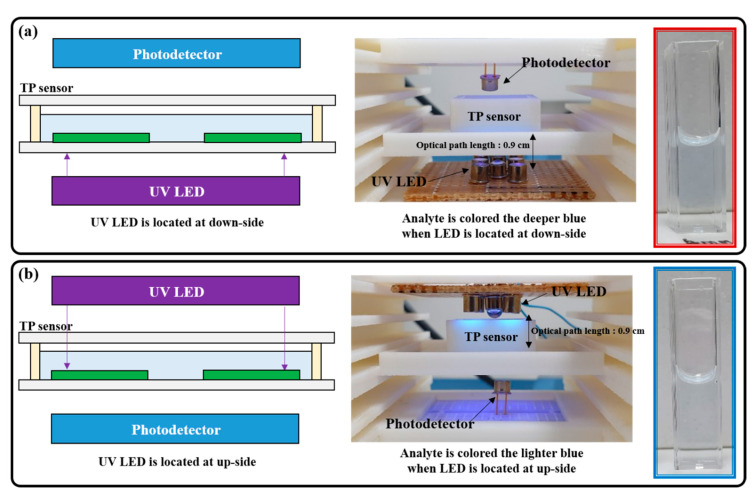
Schematic of the experimental setup and picture of the colored analytes along the UV LED locations: (**a**) down and (**b**) up sides.

The pretreatment processes were conducted on the miniaturized portable device by changing the UV LED array locations and the operating current to characterize the relationship between the UV light intensity and the efficiency of the photocatalytic-reaction-based pretreatment process, as shown in Figure 10.

The pretreatment process based on the photocatalytic reaction mainly used hydroxyl radicals (OH·) with excellent oxidation strength. The hydroxyl radicals were generated by the reaction of the generated EHPs and the hydroxyl ions (OH^−^) in water. EHPs were generated at the photocatalytic (TiO_2_) surface during the UV light illumination. Therefore, the loss of the UV light reaching the photocatalytic surface must be minimized to improve the pretreatment efficiency based on the photocatalytic reaction. Water absorbs a small amount of UV light; thus, we investigated the pretreatment efficiency along with the UV LED array location first, before the UV light intensity was changed, by increasing the operating current.

The pretreated analyte was colored a deeper blue when the UV LED was located at the down side (i.e., the optical path length was equal in both down and up sides). This minimized the loss of light reaching the photocatalyst layer because the UV light emitted from the UV LED array did not pass through the analyte. All pretreatment processes utilizing the miniaturized TP analysis device will be conducted by installing the UV LED at the down side.

The efficiency of the photocatalytic-reaction-based pretreatment process was investigated as a function of the UV light intensity (we referred to the manufacturer’s manual for the range of UV LED operating current) by changing the UV LED array operating current, with a range from 40 mA to 100 mA. The change of the UV light intensity was confirmed by measuring the output current of the photodetector. The output current of the photodetector was generally increased by the increasing number of generated electrical carriers as the stronger UV light reaches the photodetector. We confirmed that the output current of the photodetector is increased from 0.341 to 0.509 μA by increasing the UV LED array operating current from 40 to 100 mA.

Before the miniaturized portable TP analysis device was characterized as a function of the UV light intensity, analytes with various TP concentrations were pretreated using the autoclave and colored blue with a prepared colorizing agent. These analytes were then utilized as the reference analytes.

**Figure 11:**
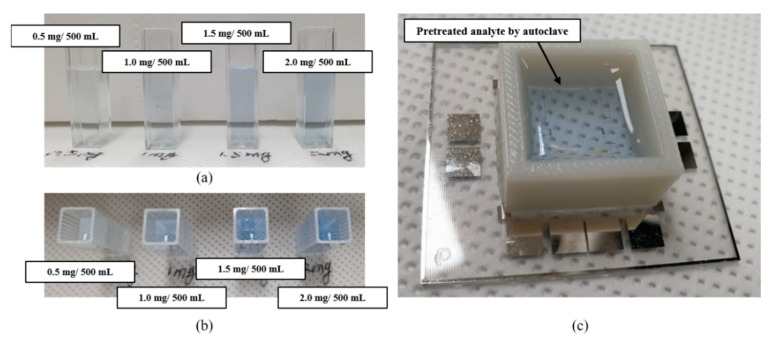
Autoclave-pretreated and blue-colored analytes with various TP concentrations: (**a**) cross view and (**b**) top view; (**c**) analyte transferred to the fabricated TP sensor for the blue-colored analyte absorbance measurement.

The analytes used as references (0.5 mg/500 mL, 1.0 mg/500 mL, 1.5 mg/500 mL, and 2.0 mg/500 mL) were pretreated for 30 min under the conditions of 122.6 °C temperature and 1.19 kg·cm^−2^ pressure using the autoclave. The analytes pretreated by the autoclave were transferred to the fabricated TP sensor. Subsequently, a prepared colorizing agent was injected into the transferred analyte. Through these procedures, optical properties of transferred and blue-colored analytes were analyzed by measuring the electrical signal of the photodetector, which is installed in the miniaturized portable TP analysis device. The procedure of transferring the analyte can be omitted when the photocatalytic-reaction-based pretreatment method is utilized. This means that the entire process for TP analysis can be easily conducted on the miniaturized portable TP analysis device, sequentially. The pretreated analyte was colored a deeper blue by increasing the TP concentration, as shown in Figure 11. The TP concentration was analyzed by quantitatively measuring the output current of the photodetector installed in the miniaturized portable TP analysis device.

**Figure 12:**
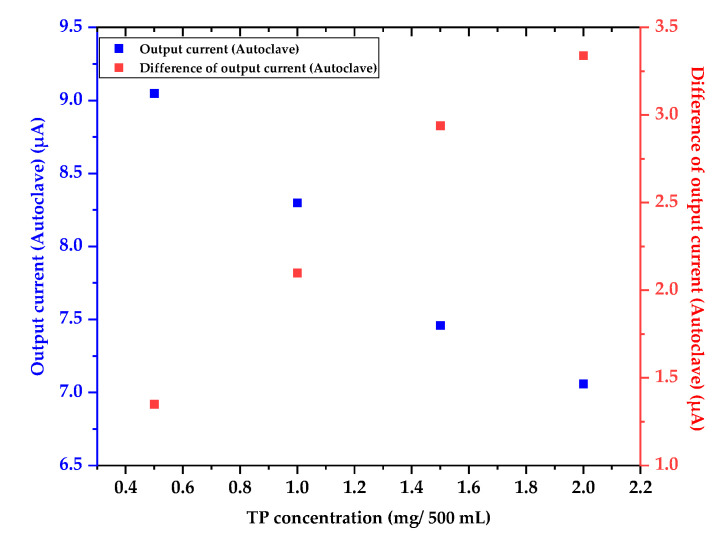
Graph of the output current and the difference of the output current as a function of the TP concentration (autoclave).

The blue-colored analytes had a maximum absorption wavelength of 880 nm. The output current and the difference of the output current were measured by utilizing the LED-emitting light with an 880 nm wavelength and the photodetector with a measurable range of 300 to 1100 nm, as shown in Table 1 and Figure 12. 

The color of the pretreated analytes was a deeper blue with the increasing TP concentration; hence, the output current of the photodetector decreased, and the difference of the output current increased. That is, the analytes with a deeper blue color had a higher TP concentration, and TP concentration can be analyzed by estimating the absorbance of the blue-colored analytes. Output current and difference of the output current of the analytes pretreated by the autoclave were used as the reference data for characterizing and optimizing the miniaturized portable TP analysis device. 

**Figure 13:**
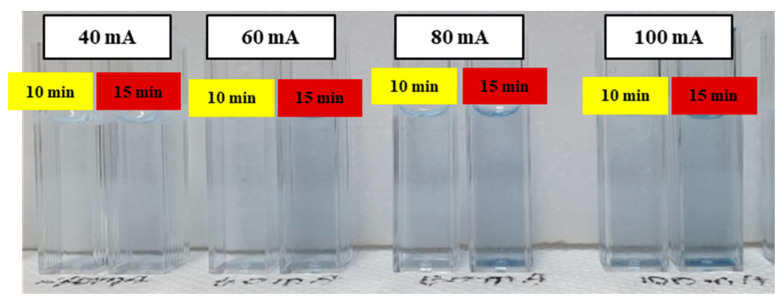
Pretreated and blue-colored analytes (2 mg/500 mL) as functions of the pretreatment time and the UV LED array operating current.

**Figure 14:**
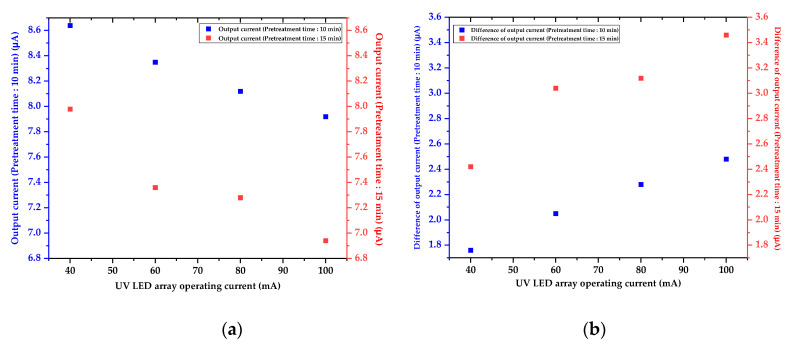
(**a**) Graph of the output current as functions of the pretreatment time and the UV LED array operating current and (**b**) graph of the difference of the output current as a function of the pretreatment time and the UV LED operating current.

The analytes with 2 mg/500 mL TP concentration were pretreated utilizing the miniaturized portable TP analysis device, changing the pretreatment time and the UV LED operating current. The pretreated analytes were colored blue under the various pretreatment conditions, as shown in Figure 13. The pretreatment efficiency was estimated by measuring the absorbed light intensity, and the obtained results were compared with the pretreatment efficiency of the autoclave. The experimental results denoted that the photocatalytic reaction was more actively generated by increasing UV light intensity; thus, the efficiency of the photocatalytic-reaction-based pretreatment process was also dramatically improved. In Table 2 and Figure 14a,b, the measured output current and the difference of the output current of the pretreated analytes for 15 min and under 100 mA UV LED operating current show characteristics similar to those of the conventional TP pretreatment equipment (i.e., autoclave). In other words, the analytes containing phosphorus could be pretreated and analyzed under conditions of room temperature, atmospheric pressure, and a shortened analysis time when the analytes are pretreated using the miniaturized portable TP analysis device.

**Figure 15:**
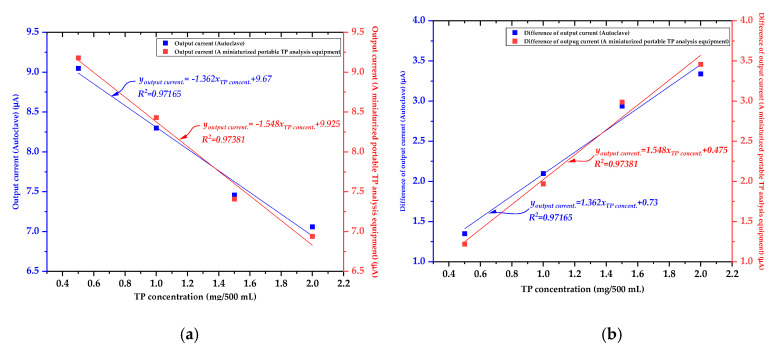
(**a**) Graph of the output current as a function of the TP concentration utilizing the autoclave and miniaturized TP analysis system (pretreatment conditions: 100 mA UV LED array operating current and 15 min pretreatment time) and (**b**) graph of the difference of the output current as a function of the TP concentration utilizing the autoclave and the miniaturized TP analysis system (pretreatment conditions: 100 mA UV LED array operating current and 15 min pretreatment time).

Finally, the analytes with various TP concentrations (i.e., 0.5 mg/500 mL, 1.0 mg/500 mL, 1.5 mg/500 mL, and 2.0 mg/500 mL) were pretreated by utilizing the miniaturized portable TP analysis device with optimized pretreatment conditions (i.e., 100 mA UV LED array operating current and 15 min pretreatment time) and conventional pretreatment equipment (i.e., autoclave). Their absorbance was measured by using the LED and the photodetector, respectively. Table 3 and Figure 15 show the measured output current and the difference of the output current as a function of the TP concentration when the same analytes were pretreated using the autoclave and the miniaturized portable TP analysis device, respectively. 

Measured output current and difference of output current as a function of TP concentration were similar for both the miniaturized portable TP analysis device (100 mA of UV LED array operating current, 15 min of pretreatment time) and autoclave (122.6 °C of pretreatment temperature, 116.9 kPa of pretreatment pressure). The equations between the measured output current and difference of output current as a function of variations in TP concentration were derived as follows:

$$y_{\mathrm{output}\;\mathrm{current}.}=-1.548x_{\mathrm{TP}\;\mathrm{concent}.}+9.925\;\left(\mathrm{by}\;\mathrm{miniaturized}\;\mathrm{TP}\;\mathrm{analysis}\;\mathrm{system}\right)$$

$$y_{\mathrm{output}\;\mathrm{current}.}=-1.362x_{\mathrm{TP}\;\mathrm{concent}.}+9.67\;\left(\mathrm{by}\;\mathrm{Autoclave}\right)$$

$$y_{\mathrm{Difference}\;\mathrm{of}\;\mathrm{output}\;\mathrm{current}.}=1.548x_{\mathrm{TP}\;\mathrm{concent}.}+0.475\;\left(\mathrm{by}\;\mathrm{miniaturized}\;\mathrm{TP}\;\mathrm{analysis}\;\mathrm{system}\right)\;$$

$$y_{\mathrm{Difference}\;\mathrm{of}\;\mathrm{output}\;\mathrm{current}.}=1.362x_{\mathrm{TP}\;\mathrm{concent}.}+0.73\;\left(\mathrm{by}\;\mathrm{Autoclave}\right)$$

Therefore, an analyte containing unknown TP concentration could be easily and simply analyzed by deriving the relationship between the measured difference of output current (or output current) and TP concentration by using the miniaturized portable TP analysis device, which has a relatively linear relationship between the measured output signal and TP concentration (R^2^ = 0.97381). 

The experiment results show that a miniaturized portable TP analysis device could replace the conventional TP analysis system under conditions of room temperature and atmospheric pressure, even with a shortened analysis time.

## 5. Conclusions

In this paper, a photocatalytic-reaction-based TP sensor with a small size is proposed and fabricated, and a miniaturized portable TP analysis device is configured by utilizing an LED array, photodetector, and the fabricated TP sensor. The existing TP analysis system is only used in the laboratory because of its bulky size, expensiveness, and long analysis time. For this reason, it is very difficult to prevent eutrophication in advance and monitor TP concentration in real time. As aquatic ecosystem pollution (including algal bloom and eutrophication, which deprive fish of oxygen) is accelerated by industrialization, urbanization, and fertilizer overuse, there has been a growing need for the development of a portable water quality monitoring system. The miniaturized portable TP analysis device in this paper utilizes a pretreatment method with photocatalytic reaction. Because the pretreatment method with photocatalytic reaction is conducted in room temperature and atmospheric pressure, it provides freedom in the design and fabrication of the proposed portable equipment. Therefore, all TP analysis procedures which consist of pretreatment and measurement could be realized on a miniaturized portable TP analysis device by utilizing MEMS, LOC, and 3D printing technologies. To enhance the efficiency of the photocatalytic reaction, there are various methods, such as supplying thermal energy and electron scavengers, increasing the photocatalytic area and UV light intensity, and so on. From among these methods, increases in UV light intensity were used to improve the efficiency of the photocatalytic reaction in this paper. After pretreatment based on photocatalytic reaction is conducted on the miniaturized portable TP analysis device, the TP concentration is quantitatively measured by estimating the absorbance of blue-colored analyte as a function of TP concentration. To estimate the absorbance of blue-colored analyte, LED lights with a peak wavelength of 880 nm and a photodetector are located at the down-side and up-side of the fabricated TP analysis system, respectively. The equipment can obtain a wide linear response range from 0.5 mg/L to 2.0 mg/L (R^2^ = 0.97381), and its performance can be improved by increasing the intensity of the UV light emitted from the LED array. Finally, results of TP analysis are similar to those of the conventional TP pretreatment equipment (autoclave) when a UV LED operating current of 100 mA is applied to the miniaturized portable TP analysis device for 15 min. Although these achievements utilizing a miniaturized portable TP analysis device are encouraging, there are still some challenges for on-site and real-time TP measurement. As mentioned above, phosphorus exists in diverse forms (only sodium glycerophosphate (C_3_H_7_Na_2_O_6_P) is employed as the phosphorus solution in this study) in unknown water environments, and this may be a restraint for accurate TP analysis. Therefore, in order to apply this device to the on-site and real-time TP analysis fields and substitute conventional TP analysis equipment, phosphorus which exists diverse forms in unknown water environments should be considered. In future works, the miniaturized portable TP analysis device will be characterized in terms of its TP pretreatment and measurement performances when diverse forms of phosphorus solutions are used as analyte, or even real water samples. Then, the readout-integrated circuit (ROIC) which operates the LED and photodetector will be designed, fabricated, and integrated into the miniaturized portable TP analysis device. Therefore, the miniaturized portable TP analysis device in this paper is a potential portable candidate for on-site and real-time TP monitoring systems, without time and location constraints and harsh operating conditions such as high temperature and internal pressure.

## Figures and Tables

**Table 1:** Output current and difference of the output current as a function of the TP concentration of the analytes pretreated by the autoclave (i.e., the difference of the output current was calculated by differencing the output current between the DI (without phosphorus) and the analytes (with phosphorus)).

TP Concentration	Output Current (μA)	Difference of Output Current (μA) (Ref. Deionized Water)
**Air**	11.5	
**Only TP sensor**	10.8	
**Deionized water**	10.4	0
**0.5 mg/500 mL**	9.05	1.35
**1.0 mg/500 mL**	8.30	2.1
**1.5 mg/500 mL**	7.46	2.94
**2.0 mg/500 mL**	7.06	3.34

**Table 2:** Output current and difference output current as a function of the pretreatment time and the UV LED operating current. (The TP concentration of the used analyte was 2 mg/500 mL. The difference of the output current was calculated by differencing the output current between the DI (without phosphorus) and the analytes (with phosphorus).)

		* Output Current (μA)		** Difference of Output Current (μA)	
	Pretreatment Time	10 min	15 min	10 min	15 min
UV LED Operating Current					
**40 mA**		8.64	7.98	1.76	2.42
**60 mA**		8.35	7.36	2.05	3.04
**80 mA**		8.12	7.28	2.28	3.12
**100 mA**		7.92	6.94	2.48	3.46

* Output current (μA): 11.5 (Air), 10.9 (Only TP sensor), 10.4 (DI), 7.06 (Autoclave), ** Difference of output current (μA): 3.34 (Autoclave).

**Table 3:** Comparison of the autoclave and the miniaturized TP analysis system (pretreatment conditions: 100 mA UV LED array operating current and 15 min pretreatment time). The difference of the output current was calculated by differencing the output current between the DI (without phosphorus) and the analytes (with phosphorus).

		* Output Current (μA)		Difference of Output Current (μA)	
	Used Equipment	** Autoclave	*** Miniaturized Portable TP Analysis Device	** Autoclave	*** Miniaturized Portable TP Analysis Device
TP Concentration					
**0.5 mg/500 mL**		9.05	9.18	1.35	1.22
**1.0 mg/500 mL**		8.30	8.43	2.1	1.97
**1.5 mg/500 mL**		7.46	7.41	2.94	2.99
**2.0 mg/500 mL**		7.06	6.94	3.34	3.46

* Output current (μA): 11.5 (Air), 10.9 (Only TP sensor), 10.4 (DI); ** Pretreatment condition (Autoclave): 122.6 °C of pretreatment temperature, 116.9 kPa of pretreatment pressure, 30 min of pretreatment time; *** Pretreatment condition (Miniaturized portable TP analysis device): Room temperature, atmospheric pressure, 15 min of pretreatment time.

## Data Availability

The data presented in this study are available on request from the corresponding author.
